# Subjective well-being among Latino day laborers: Examining the role of religiosity, social networks, and cigarette use

**DOI:** 10.15171/hpp.2018.06

**Published:** 2018-01-07

**Authors:** Javier F. Boyas, Pamela Valera, Erika Ruiz

**Affiliations:** ^1^Department of Social Work, School of Applied Sciences, University of Mississippi, University, MS, USA; ^2^Department of Social and Behavioral Health Sciences & Department of Urban Health Administration, School of Public Health, Rutgers University, Newark, NJ, USA; ^3^School of Social Work, University of Texas at Arlington, Arlington, Texas 76016, USA

**Keywords:** Latino day laborers, Subjective well-being, Undocumented status, Cigarette use

## Abstract

**Background:** Latino day laborers (LDLs) experience chronic stressors, that adversely affects their subjective well-being (SWB). The purpose of the study was to determine whether LDLs’ sociodemographic characteristics, religiosity, social networks, and cigarette use were significantly associated with SWB.

**Methods:** AApproximately 150 LDLs from 4 informal day laborer sites in Dallas and Arlington, Texas, participated in the cross-sectional survey. Informed consent was obtained from all participants and data on SWB were collected face-to-face from April 2013 through July 2013.

**Results:** Most respondents were of Mexican ancestry, (n = 112; 75%), were foreign-born (n = 140; 93%), and undocumented (n = 108; 72%). LDLs reported a mean satisfaction with life score of 19.07 (SD=8.52). Thirty percent of LDLs reported not smoking any cigarettes in the past 30 days. However, 20% reported smoking 20–39 cigarettes, while another 20% reported smoking 40 or more cigarettes in the past 30 days. Hierarchical multiple regression results indicated that marital status (β = 0.13, P ≤ 0.05), connectedness to friends (β = 0.21, P ≤ 0.001), the influence of religion on life (β = 0.57, P ≤ 0.01), frequency of attendance to religious institution (β = 0.11, P ≤ 0.005), and cigarette use (β = -0.15, P ≤ 0.05) were significantly associated with the SWB appraisals of LDLs.

**Conclusion:** These findings show that LDLs are resilient and rely on noneconomic factors to enhance their perceived quality of life. The results underscore the need to identify pathways to improve SWB among LDLs. Doing so may address the broader mental health and occupational health disparities gaps that affect LDLs.

## Introduction

The only national study on day laborers estimated that there are at least 117,647 day laborers in the United States, of whom over 90% are Latino immigrants.^1^ Given the temporary nature of their work, Latino day laborers (LDLs) experience poor social determinants of health and suffer poor physical and mental health outcomes in part because day labor employment does not provide high pay, health insurance, or steady income.^1-3^ This exacerbates the chronic stressors experienced by LDLs, such as fears of deportation, economic insecurity, occupational risks, and dire working conditions, and often results in poor health outcomes.^4,1^

A limitation of the current literature on LDLs is that much of it has focused predominately on pathology and risk, which neglects the exploration of positive functioning as it relates to mental health.^5^ Perceived satisfaction with life, which is a function of subjective well-being (SWB), could be a protective factor against poor health and mental health outcomes, discrimination, and uncertainty among LDLs.^6^ For this study, SWB refers to a person’s subjective assessment of how they feel in relation to their current state of living.^6^ It is believed that self-assessments of well-being can be a useful indicator for examining the psychological consequences of the lived experiences of a specific group because the construct offers an ongoing evaluation and reaction to one’s life.^7^ The strength of SWB is the notion that individuals are able to reflect on a person’s life and thus gives precedence and value to his or her lived experiences.^8^

Another limitation of the existing literature is that research on LDLs has shown that social isolation may encourage several health-compromising behaviors, such as crack use,^9^ alcohol use,^10^ and solicitation of sex with sex workers.^11,12^ However, minimal attention has focused on cigarette use. There is some evidence suggesting that a number of LDLs may turn to smoking cigarettes to cope with daily stressors, given that smoking is associated with somatic feelings of relaxation from the body’s reaction to stress. One study indicated that 60% of LDLs with high stress levels reported increased cigarette use in their lifetime.^13^ Additionally, construction work, which is the trade of the majority of the LDLs, has been identified as having the highest prevalence of smoking patterns compared with the general population.^14^ That prevalence is in part because clean indoor air laws do not apply to construction workers working outdoors, where it is easier to smoke.^15^ Thus, smoking behaviors should be a topic of concern among LDLs.

The literature suggests that a number of factors contribute to an individual’s response to SWB. Subjective measures of well-being evaluate a person’s or group’s level of satisfaction with their life expectations compared with their objective realities. Assessing levels of SWB can solicit a response that assesses multiple dimensions of a person’s life. It could include macro-level social conditions that relate to the environment in which they live,^16^ as can micro-level experiences, such as living in poverty or homelessness.^17^ If those living expectations far exceed their current living conditions, their subjective evaluation of their real-life situation is expected to diminish. SWB is an important construct to consider given that researchers have identified it as a factor associated with health and longevity.^18^ The broader SWB literature suggests that: independent factors, such as sociodemographic characteristics (age,^19^legal status,^17^ education,^20,21^ income,^22,23^ and marital status,^24,25^), religiosity,^2,5,26^ social networks,^2,27^ acculturation,^28,29^ and cigarette use,^30-33^ are significantly associated with SWB. The majority of these SWB studies did not exclusively focus on LDLs.

### Present Study 

Using a socioecological framework, the purpose of this study was to identify individual and micro system influences that affect SWB among LDLs. The focus of the analysis was limited to the micro system, given that in general Latinos are richer in various forms and types of resources at the individual level, but not so much at the larger, macro level.^34,35^ The present study sought to establish two objectives. The first objective was to determine whether sociodemographic characteristics, acculturation, religiosity, and social network significantly predict SWB among LDLs. The second objective was to determine whether smoking behavior predicted SWB after controlling for sociodemographic characteristics, acculturation, religiosity, and social network. Based on existing literature on SWB, it hypothesized that SWB would be higher among LDLs who: are older, have legal status, were married or partnered, reported higher levels of education and income, more acculturated, more religious, more socially connected, and reported lower levels of cigarette use compared with LDLs who were younger, undocumented, not partnered or married, reported lower levels of education and income, were less acculturated, less religious, less socially connected, and reported increased cigarette use.

## Materials and Methods

### Research design

This exploratory study used a cross-sectional research design. A convenience sample of LDLs (N = 150) was street-recruited to participate in this study. The sample size is consistent with other studies that have focused on LDLs.^4,5,36^ The study inclusion criteria were (1) self-identify as either Hispanic or Latino, (2) be at least 18 years of age, and (3) be actively seeking informal or short-term contingent employment.

### Procedures 

Data were collected face-to-face from April 2012 through July 2012 from 4 popular informal day laborer sites in Dallas and Arlington, Texas. The first author and bilingual/bicultural research assistants carried out the data collection procedures. To enhance the reliability of data collection, all research assistants were trained on culturally appropriate interviewing skills and were asked to familiarize themselves with each item of the survey. The survey questionnaire was translated to Spanish using procedures outlined by Marin and Vanoss Marin.^37^ Specifically, the research team members first carried out all translations independently. Next, the group consensually modified the survey items to best meet linguistic and cultural equivalence. Pilot testing with 25 LDLs in Arlington was conducted to further maximize the face validity of the questionnaire. Their responses were not included in the current analysis.

Surveys were self-administered, but an option of having the research assistants read the survey verbatim was offered to all participants to ensure that respondents with literacy and comprehension concerns were not discouraged from participating. Roughly 40% of the participants requested to have the survey read aloud to them. No statistically significant differences in SWB were identified between respondents who had the survey read to them compared with those who completed the survey on their own. Each participant took between 45 and 50 minutes to complete the survey. Written informed consent was obtained from all participants. Participants were compensated with $20.

### Instruments

*Dependent variable*

The life satisfaction index dependent variable was a sum of 5 single items. Each item was measure on a 7-point Likert scale with responses ranging from 1 (*strongly disagree*) to 7 (*strongly agree*). A sample item includes, “The conditions of my life are excellent.” The life satisfaction index had acceptable internal consistency (Cronbach α = 0.81).

*Sociodemographic variables*

Age was a single, continuous variable. Marital status was measured as a single, dichotomized item: 1 = married or partnered; 0 = not partnered or married. Education was dichotomized: 1 = beyond high school diploma; 0 = high school diploma or less. Income was a single, continuous item but was dichotomized because of lack of variation: 1 = 2011 income greater than $20 000; 0 = 2011 household income lower than $20 000. Legal status was a single, dichotomized item: 1= American citizen or legal resident; 0 = undocumented.

*Acculturation variable*

The Short Acculturation Scale for Hispanics for use with the Latino population was used.^38^ Respondents were asked about what language they read and speak, what language they usually speak at home, in which language they usually think, and what language they speak with their friends. These questions were answered on a 5-point Likert-type scale, including the following categories: 1 = *only Spanish* to 5 = *only English*. In the present study, the internal consistency for this scale was Cronbach α = 0.90, with higher scores representing higher levels of acculturation.

*Religiosity*

Three single items were used to determine LDLs connection to religion.^39^ Respondents were asked, “How religious are you?”, “How much influence does religion have on your life?”, and “How often do you attend church?” The first 2 items were measured on a 4-point Likert-scale that ranged from 1 = *not at all* to 4 = *very much*. The question on the frequency of attending church had possible responses ranging from 1 = *never*; 2 = *once or twice a year*; 3 = *once every 2 or 3 months*; 4 = *once in a month*; 5 = *2 or 3 times a month*; 6 = *once a week or more*.

*Social network*

The Lubben Social Network Scale,^40^ consisting of 6 items, was used to determine social engagement with one’s social network. Three items measured engagement with family, while another 3 items measured engagement with friends. Each item was measured on a 5-point Likert scale with responses ranging from 0 = *none* to 5 = *9 or more times*. A sample item includes, “How many relatives do you see or hear from at least once a month?” The social network scale had acceptable internal consistency (Cronbach α = 0.85).

*Cigarette use*

A single item was used to determine smoking behavior. Respondents were asked to state, “How many times have you smoked cigarettes in the last 30 days?”

### Analytic strategy

Univariate statistics were used to discern the study sample in terms of sociodemographic characteristics. Bivariate analyses included Pearson *r* zero-order correlations to determine whether multicollinearity was an issue and to identify the strength between SWB and independent variables. Regression diagnostics were performed to ensure that the regression underlying assumptions were not violated.^41^ A hierarchical regression analysis was conducted to determine whether smoking was a significant predictor of SWB and whether it accounts for unique variance in SWB not accounted for by sociodemographic, religiosity, and social network. In the hierarchical regression analysis, the model was entered in 3 blocks. The “Enter” method was used in all 3 blocks. In block 1, sociodemographic characteristics were included. In block 2, acculturation, social network, and religiosity items were added, while cigarette smoking was added in block 3. Since sociodemographic variables have lower predictive value, they were entered together first and smoking was entered last since that is the variable of most interest. The bivariate and multivariate results in both tables were presented by reporting the findings sequentially in the order in which they were entered in the blocks. For example, the results of the sociodemographic variables were reported first since they were entered in block 1. Statistical significance was measured at the 95% confidence interval level (*P* ≤ 0.05). All statistical analyses were conducted using IBM SPSS version 22.0.^42^

## Results

Table 1 shows the descriptive statistics of the variables used in the analysis. Univariate statistics revealed that most respondents were of Mexican ancestry, (n = 112, 75%), with a median age of 42 years. The majority of LDLs were foreign-born (n = 140, 93%) and undocumented (n = 108, 72%). Foreign-born LDLs reported being in the United States a median of 10 years. Most of the respondents reported having less than an elementary school education (n = 86, 57%). The majority of respondents earned less than $20 000 in 2011 (n = 123, 82%). LDLs reported a mean satisfaction with life score of 19.07 (SD = 8.52). Thirty percent of LDLs reported not smoking any cigarettes in the past 30 days. However, 20% reported smoking 20–39 cigarettes, while another 20% reported smoking 40 or more cigarettes in the past 30 days.

Results of the Pearson’s *r* suggest that several variables were significantly correlated with SWB (see Table 2). Legal status was inversely associated with SWB (*r* = -0.18, *P *≤ 0.05). LDLs who held legal status reported lower levels of SWB. LDLs who were married or partnered also reported higher levels of SWB (*r *= 0.42, *P *≤ 0.001). This was also the case for acculturation. LDLs who were more acculturated reported significantly lower levels of SWB (*r *= -0.14, *P *≤ 0.001). Social engagement with friends was also positively associated with higher levels of SWB (*r *= .55, *P *≤ 0.001). Social engagement with family also shared a significant and positive relationship with SWB (*r *= 0.54, *P *≤ 0.001). LDLs who reported that religion has an influence in their daily lives also reported higher levels of SWB (*r *= 0.70, *P *≤ 0.001). Smoking cigarettes was statistically significant and negatively associated with SWB (*r *= -0.57, *P *≤ 0.001). At the bivariate level, age, income, education, how often one attends their religious institution, and how religious one is were not significantly related to SWB.

The hierarchical multiple regression showed that at block 1, of the sociodemographic characteristics, only marital status (β = 0.42) contributed significantly to the regression model, (*P *< 0.001) and accounted for 9.3% of the variance SWB (see Table 3). Introducing acculturation, social engagement with friends (β = 0.21), how often one attends their religious institution (β = 0.11), and the influence of religion (β = 0.48) variables explained an additional 24% of variation in SWB in block 2, and this change in *R^2^* was significant (*P *< 0.001). In block 2, marital status remained statistically significant. Finally, adding smoking behavior (β = -0.15) to the model explained an additional 1.8% of the variation in SWB, and this change in *R^2^* was significant (*P *< 0.001). In model 2, 5 independent variables were significant in the overall regression model and accounted for 38.2% of the variance in SWB.

## Discussion

The present study sought to investigate what factors predicted SWB among LDLs. To date, only two studies have explored how LDLs appraise their levels of SWB. Consistent with existing studies,^5,36^ our findings show that LDLs were slightly dissatisfied with their lives as a whole. Given the adverse social conditions, economic distress, and social injustice LDLs endure, it might be expected that they have greater dissatisfaction with life.^17^ However, Diener and Biswas-Diener^43^ suggest that Latinos express higher levels of happiness because of cultural values, where positive emotions are consciously more highly regarded as opposed to unpleasant emotions. These cultural groups are viewed as holding a more optimistic disposition in which they place greater weight on the pleasant events in their lives and focus less on negative circumstances.^43^

Diner et al^8^ also suggest that SWB is greater among Latinos because of its collectivist perspective, which stems from traditional forms of family composition.^44^ Latinos, in general, can be characterized by intimate and interdependent social relationships. Their households are embedded in a complex extended network of families and friends that places a stronger emphasis on social connectedness and cohesiveness.^44^ This was evident in the present study that shows that LDLs who reported significantly higher levels of SWB were respondents who were married and who were more socially involved with their friends. Being married and more socially connected to friends is more likely to broaden social networks because they pool together their network of friends, families, and contacts. It could be suggested that such level of connectedness to other LDLs may act as a mediating factor in the relationship between economic distress and SWB. Hence, a more appropriate framework to view SWB among LDLs would be through a human nature approach in which an individual’s needs are assessed through a web of social relationships that can provide ongoing support.^43^ This would further explain why socioeconomic indicators, such as income and education, have little predictive value when examining SWB among LDLs.

The results show partial support for the specified hypotheses. Surprisingly, legal status significantly predicted SWB; US citizens reported significantly lower levels of SWB than noncitizens. The majority of LDLs in the present study were foreign-born, which may partially explain this finding. In cross-national studies of SWB, citizens from Latin America were found to have higher levels of SWB, despite economic adversity.^45^ Another explanation of this finding could relate to the views and aspirations of LDLs born and reared in the US, which may be more individualistic and materialistic in nature. This outcome is further corroborated by the direction of the acculturation variable, which suggests similar results. Both variables indicate that SWB is lower among LDLs who are US citizens and are more acculturated. It is plausible that LDLs who are US citizens may have lower levels of SWB because they have to vie with undocumented workers for unstable work that is low paying. US-born LDLs may be more disillusioned and dissatisfied with life given their economic instability and lack of economic mobility.

This study did not establish that a significant relationship exists between family and SWB. This is surprising, given that family has been shown to affect SWB among LDLs.^5^ It is plausible that since many LDLs who migrate to the United States leave families in their country of origin,^2^ the lack of physical and immediate presence may have affected how much affect family had on their reported levels of SWB. It would be hard to have a close relationship with family members who are not accessible because of geographic distance. In the case of LDLs, the effects of their strong ties to other LDLs may have trumped the effects of family. Negi and colleagues^2^ found that LDLs rely heavily on other LDLs for fellowship. LDLs help each other by identifying day labor corners, warning against exploitive employers, and even transporting video letters to their country of origin, all of which facilitate increased levels of well-being.

The results of this study, like others, suggest that religion plays a major role in predicting SWB among LDLs.^2,5^ It appears that religion may promote a greater sense of resilience that may safeguard against negative experiences that adversely affect their well-being.^5^ In a qualitative study, LDLs reported that religion offered spiritual comfort and the church itself provides them with a space where they can find social and emotional support during difficult times.^2^

The present study shows that 70% of the LDLs who participated in this study smoked at least a couple of cigarettes in the previous 30 days. Moreover, the results show that increased cigarette use does significantly predict lower levels of SWB. This is consistent with other studies that have noted that smokers are less satisfied with their lives.^33,34^ This is not surprising, given that LDLs contend with many daily stressors and work mostly in an industry that is recognized as having a higher prevalence of smoking than the general population. This finding is consistent with existing research that links a stressful life, workplace stress, and nature of work as predictors of the smoking behavior of an individual.^11-13^

## Limitations

There are several limitations in this study. The cross-sectional study does not allow for any causal inferences. Second, the results have limited generalizability, given that the data were collected from only 4 sites in 2 cities in Texas, non-probability sampling methods were used, and a relatively small sample size was achieved (N**= 150). However, regarding the sample size, Crosby et al^46^ argue that research should be a balance between methodological rigor and the value of the issues being examined with a hard-to-reach population, such as LDLs. As noted by Crosby et al, “Although smaller samples may be limited in their statistical power to effectively test hypotheses and the precision of effect estimates may be less stable, a key consideration is the value of the findings in addressing gaps in the empirical literature as these gaps may be valuable for informing public health policy and practice” (p. 3). This consideration is applicable in this case because LDLs are a hidden population and many may not be willing to participate in any “traditional research” endeavor due to fears of being identified and possibly deported. Although the sample is not large, the findings are unique and add a greater understanding to the literature on SWB among LDLs. Third, a self-report survey was used to document smoking patterns. In addition, smoking behavior was measured only in the past 30 days and the single open question item is a limited measure of understanding patterns of cigarette use. We therefore lack accurate information about patterns, types of tobacco products used, and the nature of smoking behaviors. Such information would have been useful for understanding smoking behaviors in relation to SWB.

## Implications

Notwithstanding its limitations, several implications can be drawn from the findings. First, SWB among LDLs is tied closely to religious beliefs. Perhaps professionals and faith-based organizations can partner to provide services at the churches or partner with churches to provide outreach. It is likely that LDLs will make use of services if they are part of larger faith-based initiatives. Second, LDLs in the present study reported engaging in smoking behaviors that have gone overlooked by health researchers. This is a preliminary finding that warrants further research. It may be prudent for professionals to develop culturally appropriate smoking cessation programs that aim to decrease the prevalence of smoking behaviors among such a vulnerable group. Otherwise, this overlooked health-risk behavior may help contribute to the health disparities experienced by a group that is at elevated risk of contracting cancer, heart disease, and stroke, all of which are connected to smoking cigarettes and subsequent mortality.^47^ Third, given that the majority of participants in this study were undocumented immigrants, and given the current anti-immigration climate affecting undocumented Latinos, it is more important than ever to identify factors that protect LDLs’ well-being. Furman, Ackerman, and Negi^48^ provide policy and practice implications that may be useful for practitioners to consider, given that undocumented LDLs may require services in a variety of settings (e.g., hospitals, criminal justice systems, mental health agencies). Practitioners should also be aware of proposed anti-immigration state and national policies because of the negative effect they have on physical and psychological well-being.

Last, this study has significant public health implications as the findings extend beyond protective factors attributed to positive SWB, in that it provides preliminary findings to consider risk factors such as cigarette smoking. Furthermore, an understanding of coping behaviors among LDLs and their collective circle may potentially influence how practitioners conduct health promotion outreach and other health interventions. More research is needed that also explores whether cigarette use is a reaction to the daily stressors experienced by LDLs and whether its use is a conventional coping mechanism encouraged by networks in which LDLs participate. Future studies on coping strategies designed to enhance or promote SWB are urgently needed.

## Conclusion

In conclusion, the present study examined SWB among LDLs. Evaluating SWB is important, given that this indicator provides a first-hand appraisal of how LDLs view their lives. This study revealed that better life evaluations among LDLs included engaging with friends and family, religious connection, and being married or with a life partner. These findings show that LDLs are resilient and rely on noneconomic influences to enhance their perceived quality of life. This study also points to the importance of smoking behaviors as a negative predictor of SWB. The findings underscore the need to identify pathways to improve SWB among LDLs. Doing so may close the broader mental health and occupational health disparities gaps that burden the Latinos who live their lives in overlapping vulnerabilities as men of color, undocumented status, and vulnerability of their occupation.

## Ethical approval

All procedures performed in studies involving human participants were in accordance with the ethical standards of the institutional and/or national research committee and with the 1964 Helsinki Declaration and its later amendments or comparable ethical standards. Informed consent was obtained from all individual participants included in the study. The Institutional Review Board at the University of Texas at Arlington approved all protocols associated with the present study.

## Competing interests

The authors declare that they have no competing interests.

## Funding

No external funding was obtained for the current study.

## Authors’ contributions

JFB developed the concept and design of this research project; developed the manuscript; collected data, carried out the analysis and interpretation; developed the manuscript, and approved the final version of the manuscript. PV participated in the data analysis and interpretation and writing of the manuscript. ER helped in identifying measures at the beginning of the project, participated in the data collection, and contributed to the development of the manuscript. All 3 authors have reviewed, approved, and consented to the submission, and they are accountable for all aspects of its accuracy and integrity in accordance with ICMJE criteria.

## Acknowledgments

We would like to acknowledge the contributions made by the participants of the current study. The day laborers who participated could have opted out, but chose to provide us with insightful information. This was greatly appreciated.

**Table 1:** Socio-demographic characteristics of the study sample (N = 150)

**Variables and Categories**	**Mean (SD)**	**No. (%)**
Age (y)	41.97 (11.12)	
Legal status		
US citizen		10 (6.7)
Naturalized citizen		8 (5.3)
Permanent resident		24 (16.0)
Non-immigrant visa		13 (8.6)
Non-citizen, nor permanent legal resident		95 (63.4)
If Foreign-born, number of years living in the US	6.02 (5.24)	
Highest level of education achieved		
Less than elementary school		87 (58.0)
Less than high school		39 (26.0)
High school or GED		17 (11.3)
Associate’s degree		2 (0.01)
Bachelor degree		6 (4.0)
Education in native country		
Yes		125 (87.9)
No		25 (13.1)
Income		
More than $20 000 in 2011		27 (18.0)
Less than $20 000 in 2011		123 (82.0)
Married		
Yes		53 (35.0)
No		97 (65.0)
Acculturation scale	6.60 (3.44)	
Religiosity		
How much influence does religion have on your life?	3.3 (0.98)	
How religious are you?	2.25 (0.97)	
How often do you attend your religious institution?	3.05 (1.15)	
Social network		
Connectedness to relatives	6.64 (3.50)	
Connectedness to friends	8.61 (3.71)	
How many times have you smoked cigarettes in the last 30 days?		
0		45 (30)
1–2		25 (16.7)
3–5		20 (13.3)
6–9		11 (7.3)
10–19		9 (6.1)
20–39		20 (13.3)
40 or more		20 (13.3)
SWB	19.07 (8.52)	

Abbreviations: SWB, subjective well-being; SD, standard deviation.

**Table 2:** Zero-order correlation among variables

**Variables**	**1**	**2**	**3**	**4**	**5**	**6**	**7**	**8**	**9**	**10**	**11**	**12**	**13**
1. Subjective Well-Being	-												
2. Age	0.20	**-**											
3. Legal Status	-0.18^a^	0.06	**-**										
4. Education	0.08	0.17^b^	0.08	**-**									
5. Income	0.03	0.28^b^	-0.11^a^	0.12^b^	**-**								
6. Marital Status	0.43^b^	0.01	-0.15	-0.05	0.20^b^	**-**							
7. Acculturation	-0.14^a^	-0.14	0.45^b^	0.18^a^	0.12	0.21^a^	**-**						
8. How much influence does religion have on your life.	0.70^b^	0.05	-0.25^b^	-0.10^b^	0.07	-0.41^a^	-0.11^a^	**-**					
9. How religious are you?	0.02	0.17^a^	-0.07	0.05	0.10	-0.15^a^	-0.04	0.33^b^	**-**				
10. How often do you attend church?	0.05	-0.04	-0.09	-0.12^a^	-0.15^a^	-0.04	0.02	0.05	0.10	**-**			
11. Social connectedness to family	0.54^b^	0.05	-0.23^a^	0.18^b^	0.12^a^	0.23^b^	-0.22^b^	0.41^b^	0.06	0.01	**-**		
12. Social connectedness to friends	0.55^b^	0.07	0.15^b^	0.05	0.18^b^	0.24^b^	0.12^b^	0.34^b^	0.5	0.06	0.26^a^	**-**	
13. How many time have you smoked cigarettes in the past 30 days?	-0.57^b^	0.10	0.29^b^	-0.18^a^	-0.17^b^	-0.22^b^	-0.27^b^	-0.25^b^	0.06	-0.04	-0.18^a^	-.29^b^	**-**

^a^ *P* = 0.05, ^b^*P* = 0.001.

**Table 3:** Hierarchical regression model of subjective well-being among Latino day laborers

**Variable**	**B**	**Standard error B**	**β**
**Stage 1**			
Age	-0.06	0.06	-0.08
Education	0.35	0.56	-0.05
Income	-1.80	1.71	-0.08
Legal status	-2.54	1.48	-0.13
Marital status	7.33	1.34	0.42^b^
**Stage 2**			
Age	-0.01	0.04	-0.02
Education	0.04	0.39	0.01
Income	-0.78	1.110	-0.04
Legal status	-0.774	1.17	-0.04
Marital status	2.15	0.941	0.12^a^
Acculturation	0.08	0.15	0.03
Connectedness to family	0.26	0.15	0.11
Connectedness to friends	0.48	0.14	0.21^b^
How much influence does religion have in your life?	5.32	0.53	0.48^b^
How religious are you?	0.14	0.48	0.02
How often do you attend your religious institution?	-0.45	0.23	-0.11^a^
**Stage 3**			
Age	-0.01	0.04	-0.01
Education	0.14	0.38	0.02
Income	-0.78	1.09	-0.04
Legal status	0.98	1.16	0.05
Marital status	2.20	0.92	0.13^a^
Acculturation	0.11	0.15	0.05
Connectedness to family	0.09	0.17	0.04
Connectedness to friends	0.47	0.13	0.21^b^
How much influence does religion have in your life?	4.94	0.55	0.57^b^
How religious are you?	0.13	0.47	0.02
How often do you attend your religious institution?	0.45	0.22	0.11^a^
How many times have you smoked cigarettes in the last 30 days?	-0.60	0.25	-0.15^a^

Note: *R*^2^ = 9.3% for block 1, *R*^2^= 24% for block 2, *R*^2^ = 1.8% for block 3 (total R2 = 38.2%) ^a^ *P* ≤ 0.05, ^b^*P* ≤ 0.001.
